# Supplementary material for: Temperature-size responses during ontogeny are independent of progenitors’ thermal environments
Source: PeerJ. 2024 May 22;12:e17432. doi: 10.7717/peerj.17432 (PMC11127640; doi:10.7717/peerj.17432)
Supplement: Supplemental Information 1 [file peerj-12-17432-s001.docx]

**Temperature-size responses during ontogeny are independent of progenitors’ thermal environments: Supporting information**

Gerard Martínez-De León^1, *^, Micha Fahrni^1^, Madhav P. Thakur^1^

*^1^ Institute of Ecology and Evolution, University of Bern, Switzerland*

*Corresponding author

Gerard Martínez-De León

Institute of Ecology and Evolution

Baltzerstrasse 6, CH‐3012 Bern, Switzerland

Email: [gerard.martinezdeleon@unibe.ch](mailto:gerard.martinezdeleon@unibe.ch); [martinezdeleongerard@gmail.com](mailto:martinezdeleongerard@gmail.com)

**Table of content**

|  | Supplementary Methods: Experimental setup |
| --- | --- |
| Fig. S1 | Estimate of hatching success as a function of experimental temperature. |
| Fig. S2 | Distribution of the density of individuals in the experimental cohorts. |
| Fig. S3 | Egg diameter of the experimental cohorts as a function of progenitors’ temperature. |
| Fig. S4 | Egg production at first reproductive event of the experimental cohorts as a function of experimental temperature. |
| Table S1 | Output of the linear mixed effect model for the effect of the progenitors’ temperature on the adult body length of F0 individuals. |
| Table S2 | Output of the linear model for the effect of progenitors’ temperature and experimental temperature on egg development of F1 individuals. |
| Table S3 | Output of the linear mixed effect model for the effect of progenitors’ temperature on egg diameter of F1 individuals. |
| Table S4 | Output of the linear model for the effect of experimental temperature on juvenile development of F1 individuals. |
| Table S5 | Output of the linear mixed effect model for the effect of experimental temperature on mean body length at maturity of F1 individuals. |
| Table S6 | Output of quantile regression for the quantiles 0.75, 0.85 and 0.95 of the body size distribution from experimental cohorts (F1) of *Folsomia candida.* |
| Table S7 | Output of quantile regression for the quantiles 0.75, 0.85 and 0.95 of the body size distribution from experimental cohorts (F1) of *Proisotoma minuta*. |
| Table S8 | Output of the linear mixed effect model for the effect of experimental temperature on the egg diameter of F2 individuals. |
| Table S9 | Output of the linear model used for the effect of experimental temperature on egg production at first reproductive event of F1 individuals. |

**Supplementary Methods: Experimental setup**

We started the experiment by establishing populations of adult collembolans from several source cultures (i.e., progenitors; F0) in order to obtain offspring of the same age for the following phases of the experiment (henceforth referred as experimental cohorts; F1). For this purpose, we added 9 adults of *F. candida* or 11 adults of *P. minuta* into each of 80 Petri dishes (90-mm diameter) with a moist substrate of plaster of Paris and activated charcoal (9:1 mixture), in addition to dry yeast as food source provided *ad libitum*. The aim of including several F0 individuals in each plate was to obtain F1 eggs as soon as possible (e.g., within one or few days), as well as to facilitate the tracking of F1 cohorts at similar developmental stages. However, these collembolans lay egg clutches roughly once every week (Fountain and Hopkin, 2005; Tully, 2023), so it is likely that only one or a few females produced the egg clutches of the F1 generation in each plate, and thus, an equal number of offspring per female cannot be confirmed in our study. In addition, we note that it is challenging to determine how many females of the sexually reproducing *P. minuta* were included in each plate, given that the sex ratio of this species is largely unknown, including in our cultures (cf. Chahartaghi, Scheu & Ruess (2006), where the sex ratios of other Collembola species in natural conditions are described)*.* For this reason, the number of starting *P. minuta* (11 adults per plate, containing males and females in unknown proportions) was slightly higher than in *F. candida* (9 adults per plate, all parthenogenetic females), as much as the availability of adult individuals in our cultures allowed.


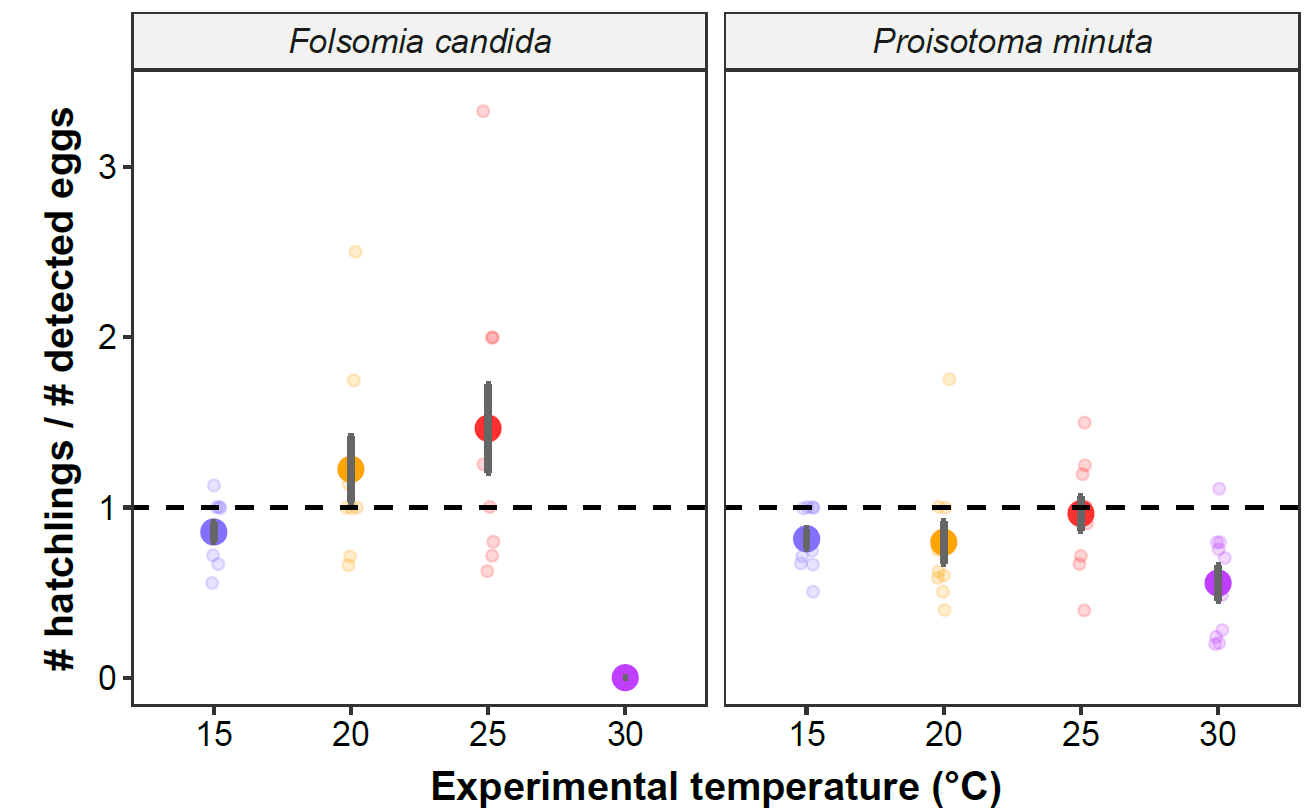


**Fig. S1.** Estimate of hatching success (number of F1 hatchlings/number of detected F1 eggs) as a function of the experimental temperature. Solid points represent means, dark bars represent standard errors, and faded points are raw data. We opted not to perform a formal analysis of this trait given that the detectability of eggs is much lower than that of hatchlings, hence there are many cases in which hatching success attains values greater than 1 (represented with a horizontal dashed line). This is because eggs are often found in holes in the substrate and aggregated in large clutches that cannot be adequately counted unless they are dissected, which may come at the risk of compromising the number of animals for further phases of the experiment if eggs are damaged during handling.


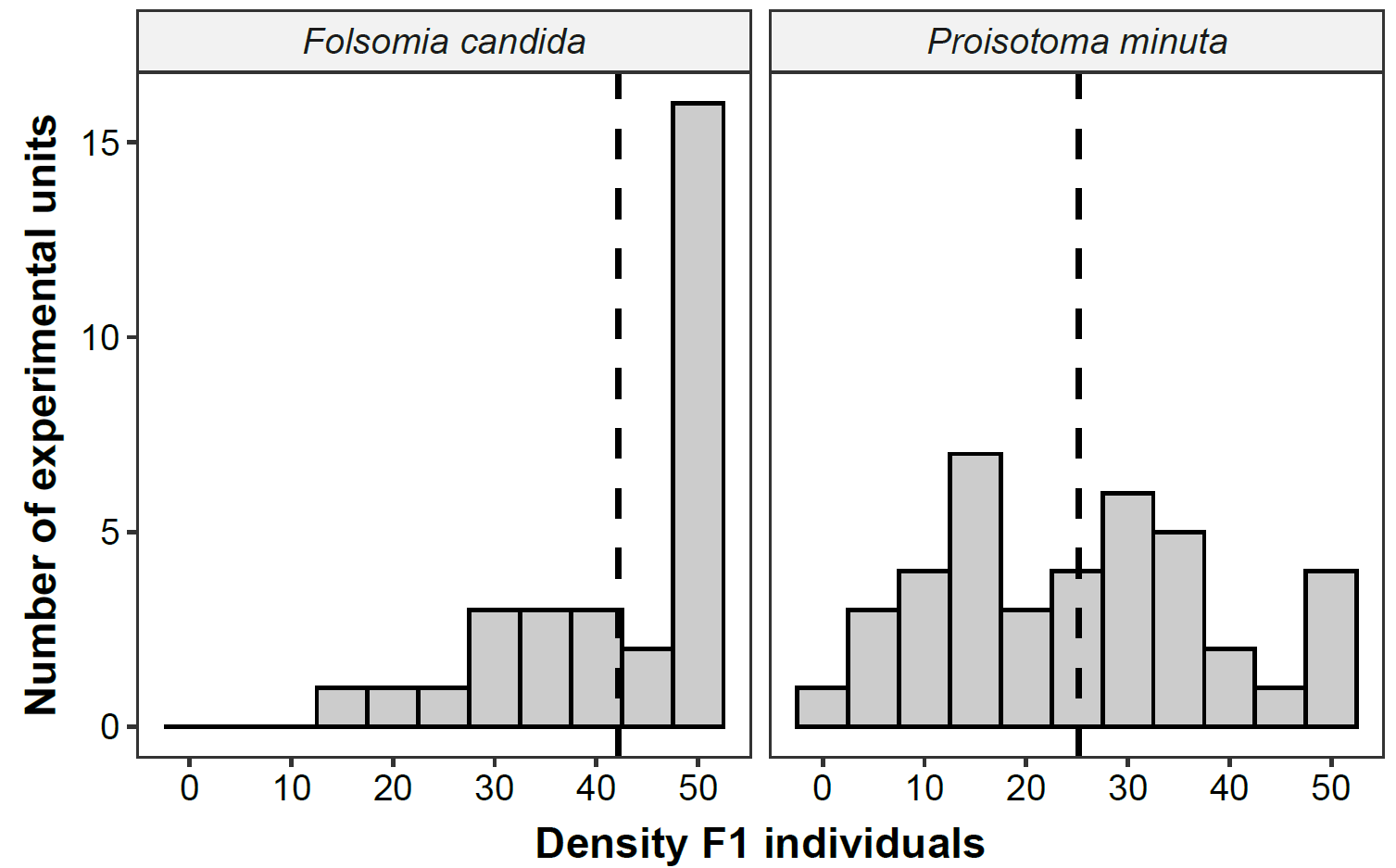


**Fig. S2.** Distribution of the density of individuals in the experimental cohorts (F1). The maximum number of individuals in each experimental unit was kept below or equal to 50, in order to control for potential effects of intraspecific competition on body sizes. The vertical dotted line represents the mean density for each species.


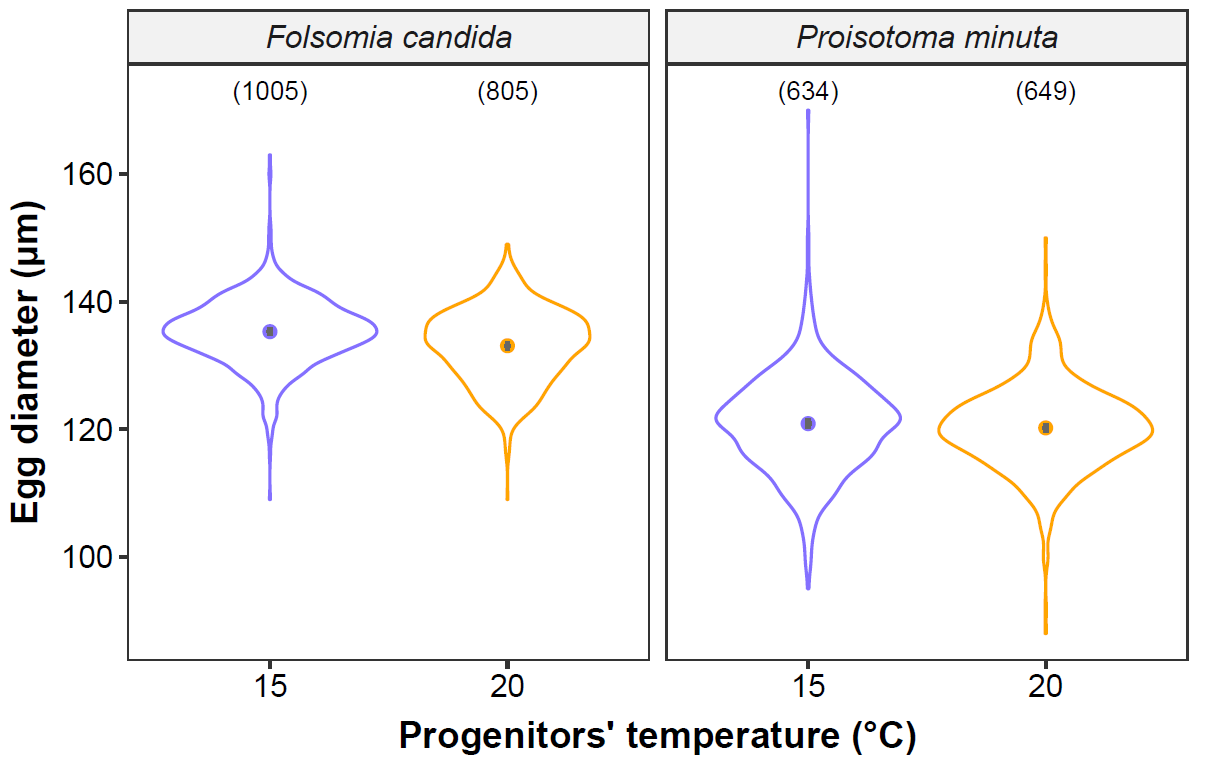


**Fig. S3.** Egg diameter of the experimental cohorts (F1) as a function of progenitors’ temperature (i.e., experienced during the lifetime of the progenitors; F0). Violin plots represent egg diameter distributions, solid points show means, and grey bars are standard errors. The number of eggs measured in each treatment is displayed in brackets. For the full model output, see Table S3.

**
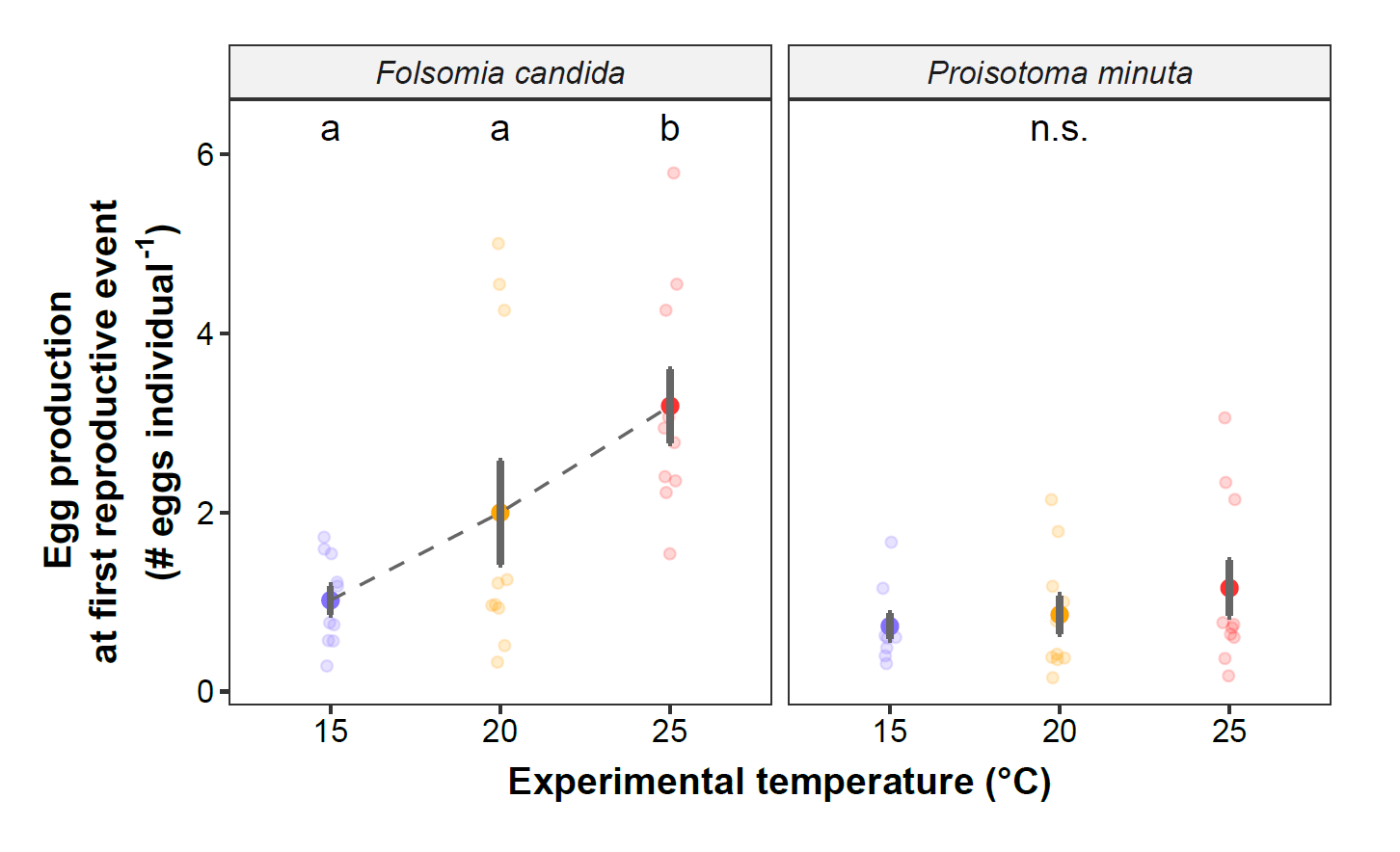
**

**Fig. S4.** Egg production at first reproductive event of the experimental cohorts (F1) as a function of the experimental temperature. Solid points represent means, dark bars represent standard errors, and faded points are raw data. Different letters indicate significant differences (*P* < 0.05) between experimental temperatures within Collembola species. For the full model output, see Table S7.

**Table S1.** Output of the linear mixed effect model used to assess the effect of the progenitors’ temperature on the adult body length of F0 individuals (see Table 1 for the model structure). We used plate ID as a random intercept to account for the dependency of individuals co-occurring in the same plate. Estimates, standard errors (SE) and p-values (*P*) of the contrasts between progenitors’ temperatures for a given species are provided, as well as the marginal and conditional R^2^. Significant p*-*values (*P* < 0.05) are highlighted in bold.

| Adult body length (µm) of the progenitors (F0) | | | | |
| --- | --- | --- | --- | --- |
| Species | Progenitors’ temperature | Estimate | SE | *P* |
| *Folsomia candida* | 15 °C | 2063.84 | 27.58 | **< 0.001** |
|  | 20 °C | 2220.69 | 27.48 |  |
| *Proisotoma minuta* | 15 °C | 1354.77 | 25.81 | 0.158 |
|  | 20 °C | 1302.34 | 26.07 |  |
|  |  |  |  |  |
| **Random effects** |  |  |  |  |
| Marginal R^2^ / Conditional R^2^ | 0.72 / 0.76 |  |  |  |
| Observations | 856 |  |  |  |
| N_groups_ (plate ID) | 80 |  |  |  |

**Table S2.** Output of the linear model used to assess the effect of the progenitors’ temperature and the experimental temperature on egg development (i.e., time until first hatching) of F1 individuals (see Table 1 for the model structure). Estimates, standard errors (SE) and adjusted p-values (*P*) of the contrasts between progenitors’ temperatures for a given species and experimental temperature are provided. Significant p*-*values (*P* < 0.05) are highlighted in bold. All pairwise contrasts between experimental temperatures were statistically significant with *P* <0.001.

| Egg development (days) of the experimental cohorts (F1) | | | | | |
| --- | --- | --- | --- | --- | --- |
| Species | Progenitors’ temperature | Experimental temperature | Estimate | SE | *P* |
| *Folsomia candida* | 15 °C | 15 °C | 16.80 | 0.25 | **<0.001** |
|  |  | 20 °C | 10.80 | 0.25 |  |
|  |  | 25 °C | 8.00 | 0.25 |  |
|  | 20 °C | 15 °C | 16.80 | 0.25 | **<0.001** |
|  |  | 20 °C | 11.20 | 0.25 |  |
|  |  | 25 °C | 8.20 | 0.25 |  |
| *Proisotoma minuta* | 15 °C | 15 °C | 14.00 | 0.25 | **<0.001** |
|  |  | 20 °C | 7.40 | 0.25 |  |
|  |  | 25 °C | 5.80 | 0.25 |  |
|  | 20 °C | 15 °C | 11.80 | 0.25 | **<0.001** |
|  |  | 20 °C | 7.20 | 0.25 |  |
|  |  | 25 °C | 5.60 | 0.25 |  |
|  |  |  |  |  |  |
|  | **Contrasts** |  |  |  |  |
| *Folsomia candida* | 15 °C vs. 20 °C | 15 °C | 0.00 | 0.35 | 1.00 |
|  |  | 20 °C | -0.40 | 0.35 | 1.00 |
|  |  | 25 °C | -0.20 | 0.35 | 1.00 |
| *Proisotoma minuta* | 15 °C vs. 20 °C | 15 °C | 2.20 | 0.35 | **< 0.001** |
|  |  | 20 °C | 0.20 | 0.35 | 1.00 |
|  |  | 25 °C | 0.20 | 0.35 | 1.00 |

**Table S3.** Output of the linear mixed effect model used to assess the effect of the progenitors’ temperature on the egg diameter of F1 individuals (see Table 1 for the model structure). We used plate ID as a random intercept to account for the dependency of eggs laid in the same plate. Estimates, standard errors (SE) and p-values (*P*) of the contrasts between progenitors’ temperatures for a given species are provided, as well as the marginal and conditional R^2^. Significant p*-*values (*P* < 0.05) are highlighted in bold.

| Egg diameter (µm) of the experimental cohorts (F1) | | | | |
| --- | --- | --- | --- | --- |
| Species | Progenitors’ temperature | Estimate | SE | *P* |
| *Folsomia candida* | 15 °C | 135.25 | 0.73 | 0.067 |
|  | 20 °C | 133.70 | 0.74 |  |
| *Proisotoma minuta* | 15 °C | 122.28 | 0.74 | 0.067 |
|  | 20 °C | 120.73 | 0.74 |  |
|  |  |  |  |  |
| **Random effects** |  |  |  |  |
| Marginal R^2^ / Conditional R^2^ | 0.51 / 0.68 |  |  |  |
| Observations | 3093 |  |  |  |
| N_groups_ (plate ID) | 80 |  |  |  |

**Table S4.** Output of the linear model used to assess the effect of the experimental temperature on juvenile development (i.e., time from first hatching until first reproduction at the plate level) of F1 individuals (see Table 1 for the model structure). Estimates, standard errors (SE) and adjusted p-values (*P*) of the contrasts between experimental are provided. Significant p*-*values (*P* < 0.05) are highlighted in bold.

| Juvenile development (days) of the experimental cohorts (F1) | | | | |
| --- | --- | --- | --- | --- |
| Species | Experimental temperature | Estimate | SE | *P* |
| Both Collembola species | 15 °C | 23.91 | 0.39 |  |
|  | 20 °C | 14.65 | 0.38 |  |
|  | 25 °C | 10.55 | 0.38 |  |
|  |  |  |  |  |
|  | **Contrasts** |  |  |  |
| Both Collembola species | 15 °C vs. 20 °C | 9.26 | 0.54 | **< 0.001** |
|  | 15 °C vs. 25 °C | 13.36 | 0.54 | **< 0.001** |
|  | 20 °C vs. 25 °C | 4.10 | 0.53 | **< 0.001** |
|  |  |  |  |  |

**Table S5.** Output of the linear mixed effect model used to assess the effect of the experimental temperature on the mean body length at maturity of F1 individuals (see Table 1 for the model structure). We used plate ID as a random intercept to account for the dependency of individuals developed in the same plate. Estimates, standard errors (SE) and adjusted p-values (*P*) of the contrasts between experimental temperatures for a given species are provided, as well as the marginal and conditional R^2^. Significant p*-*values (*P* < 0.05) are highlighted in bold.

| Body length at maturity (µm) of the experimental cohorts (F1) | | | | |
| --- | --- | --- | --- | --- |
| Species | Experimental temperature | Estimate | SE | *P* |
| *Folsomia candida* | 15 °C | 1425.33 | 19.54 |  |
|  | 20 °C | 1423.28 | 19.92 |  |
|  | 25 °C | 1404.24 | 19.88 |  |
| *Proisotoma minuta* | 15 °C | 949.65 | 20.78 |  |
|  | 20 °C | 853.57 | 20.77 |  |
|  | 25 °C | 851.67 | 20.68 |  |
|  |  |  |  |  |
|  | **Contrasts** |  |  |  |
| *Folsomia candida* | 15 °C vs. 20 °C | 2.05 | 27.90 | 0.997 |
|  | 15 °C vs. 25 °C | 21.09 | 27.88 | 0.731 |
|  | 20 °C vs. 25 °C | 19.04 | 28.14 | 0.778 |
| *Proisotoma minuta* | 15 °C vs. 20 °C | 96.08 | 29.38 | **0.005** |
|  | 15 °C vs. 25 °C | 97.98 | 29.31 | **0.004** |
|  | 20 °C vs. 25 °C | 1.90 | 29.31 | 0.998 |
|  |  |  |  |  |
| **Random effects** |  |  |  |  |
| Marginal R^2^ / Conditional R^2^ | 0.73 / 0.77 |  |  |  |
| Observations | 2193 |  |  |  |
| N_groups_ (plate ID) | 60 |  |  |  |

**Table S6.** Output of quantile regression for the quantiles 0.75, 0.85 and 0.95 of the body size distribution from the experimental cohorts (F1) of *Folsomia candida*. Sample ID was used as a random intercept to account for clustered measurements within cohorts. Estimates, bootstrapped standard errors (SE) and adjusted p-values (*P)* of the contrasts between experimental temperatures are provided. Significant p*-*values (*P* < 0.05) are highlighted in bold. Note that body size data was centred and scaled (mean = 0, standard deviation = 1) to facilitate the estimation of model parameters. Estimates may be obtained in the original scale using the following formula: original estimate = (scaled estimate x standard deviation) + mean; with mean = 1416 and standard deviation = 163.

| Quantiles of the body size distribution from the experimental cohorts (F1) of *Folsomia candida* (centred and scaled) | | | | |
| --- | --- | --- | --- | --- |
|  | Experimental temperature | Estimate | SE | *P* |
|  |  |  |  |  |
| Quantile 0.75 | 15 °C | 0.59 | 0.51 |  |
|  | 20 °C | 0.71 | 0.47 |  |
|  | 25 °C | 0.41 | 0.71 |  |
|  |  |  |  |  |
|  | **Contrasts** |  |  |  |
|  | 15 °C vs. 20 °C | -0.11 | 0.28 | 0.910 |
|  | 15 °C vs. 25 °C | 0.18 | 0.49 | 0.927 |
|  | 20 °C vs. 25 °C | 0.30 | 0.54 | 0.847 |
|  |  |  |  |  |
| Quantile 0.85 | 15 °C | 1.11 | 0.45 |  |
|  | 20 °C | 1.22 | 0.45 |  |
|  | 25 °C | 0.65 | 0.46 |  |
|  |  |  |  |  |
|  | **Contrasts** |  |  |  |
|  | 15 °C vs. 20 °C | -0.10 | 0.20 | 0.860 |
|  | 15 °C vs. 25 °C | 0.46 | 0.20 | 0.057 |
|  | 20 °C vs. 25 °C | 0.57 | 0.21 | **0.021** |
|  |  |  |  |  |
| Quantile 0.95 | 15 °C | 1.73 | 0.56 |  |
|  | 20 °C | 1.43 | 0.65 |  |
|  | 25 °C | 0.97 | 0.55 |  |
|  |  |  |  |  |
|  | **Contrasts** |  |  |  |
|  | 15 °C vs. 20 °C | 0.30 | 0.50 | 0.821 |
|  | 15 °C vs. 25 °C | 0.76 | 0.33 | 0.051 |
|  | 20 °C vs. 25 °C | 0.46 | 0.51 | 0.634 |
|  |  |  |  |  |

**Table S7.** Output of quantile regression for the quantiles 0.75, 0.85 and 0.95 of the body size distribution from the experimental cohorts (F1) of *Proisotoma minuta*. Sample ID was used as a random intercept to account for clustered measurements within cohorts. Estimates, bootstrapped standard errors (SE) and adjusted p-values (*P)* of the contrasts between experimental temperatures are provided. Significant p*-*values (*P* < 0.05) are highlighted in bold. Note that body size data was centred and scaled (mean = 0, standard deviation = 1) to facilitate the estimation of model parameters. Estimates may be obtained in the original scale using the following formula: original estimate = (scaled estimate x standard deviation) + mean; with mean = 876 and standard deviation = 150.

| Quantiles of the body size distribution from the experimental cohorts (F1) of *Proisotoma minuta* (centred and scaled) | | | | |
| --- | --- | --- | --- | --- |
|  | Experimental temperature | Estimate | SE | *P* |
|  |  |  |  |  |
| Quantile 0.75 | 15 °C | 1.19 | 0.29 |  |
|  | 20 °C | 0.61 | 0.35 |  |
|  | 25 °C | 0.44 | 0.31 |  |
|  |  |  |  |  |
|  | **Contrasts** |  |  |  |
|  | 15 °C vs. 20 °C | 0.58 | 0.27 | 0.082 |
|  | 15 °C vs. 25 °C | 0.76 | 0.29 | **0.023** |
|  | 20 °C vs. 25 °C | 0.18 | 0.37 | 0.880 |
|  |  |  |  |  |
| Quantile 0.85 | 15 °C | 1.56 | 0.58 |  |
|  | 20 °C | 1.01 | 0.67 |  |
|  | 25 °C | 0.95 | 0.51 |  |
|  |  |  |  |  |
|  | **Contrasts** |  |  |  |
|  | 15 °C vs. 20 °C | 0.55 | 0.29 | 0.149 |
|  | 15 °C vs. 25 °C | 0.61 | 0.35 | 0.194 |
|  | 20 °C vs. 25 °C | 0.07 | 0.43 | 0.987 |
|  |  |  |  |  |
| Quantile 0.95 | 15 °C | 1.72 | 0.52 |  |
|  | 20 °C | 1.91 | 0.72 |  |
|  | 25 °C | 1.28 | 0.83 |  |
|  |  |  |  |  |
|  | **Contrasts** |  |  |  |
|  | 15 °C vs. 20 °C | -0.20 | 0.46 | 0.901 |
|  | 15 °C vs. 25 °C | 0.44 | 0.48 | 0.636 |
|  | 20 °C vs. 25 °C | 0.63 | 0.77 | 0.688 |
|  |  |  |  |  |

**Table S8.** Output of the linear mixed effect model used to assess the effect of the experimental temperature on the egg diameter of the progeny (F2; see Table 1 for the model structure). We used plate ID as a random intercept to account for the dependency of eggs laid in the same plate. Estimates, standard errors (SE) and adjusted p-values (*P*) of the contrasts between experimental temperatures for a given species are provided, as well as the marginal and conditional R^2^. Significant p*-*values (*P* < 0.05) are highlighted in bold.

| Egg diameter (µm) of the progeny (F2) | | | | |
| --- | --- | --- | --- | --- |
| Species | Experimental temperature | Estimate | SE | *P* |
| *Folsomia candida* | 15 °C | 130.24 | 1.32 |  |
|  | 20 °C | 127.55 | 1.32 |  |
|  | 25 °C | 129.64 | 1.30 |  |
| *Proisotoma minuta* | 15 °C | 116.48 | 1.43 |  |
|  | 20 °C | 112.82 | 1.37 |  |
|  | 25 °C | 120.46 | 1.35 |  |
|  |  |  |  |  |
|  | **Contrasts** |  |  |  |
| *Folsomia candida* | 15 °C vs. 20 °C | 2.70 | 1.87 | 0.326 |
|  | 15 °C vs. 25 °C | 0.61 | 1.86 | 0.943 |
|  | 20 °C vs. 25 °C | -2.09 | 1.85 | 0.502 |
| *Proisotoma minuta* | 15 °C vs. 20 °C | 3.67 | 1.98 | 0.161 |
|  | 15 °C vs. 25 °C | -3.98 | 1.96 | 0.116 |
|  | 20 °C vs. 25 °C | -7.64 | 1.92 | **< 0.001** |
|  |  |  |  |  |
| **Random effects** |  |  |  |  |
| Marginal R^2^ / Conditional R^2^ | 0.39 / 0.60 |  |  |  |
| Observations | 2670 |  |  |  |
| N_groups_ (plate ID) | 59 |  |  |  |

| Egg production at first reproductive event (F1), per capita | | | | |
| --- | --- | --- | --- | --- |
| Species | Experimental temperature | Estimate | SE | *P* |
| *Folsomia candida* | 15 °C | 1.02 | 0.34 |  |
|  | 20 °C | 2.00 | 0.34 |  |
|  | 25 °C | 3.19 | 0.34 |  |
| *Proisotoma minuta* | 15 °C | 0.73 | 0.36 |  |
|  | 20 °C | 0.86 | 0.34 |  |
|  | 25 °C | 1.16 | 0.34 |  |
|  |  |  |  |  |
|  | **Contrasts** |  |  |  |
| *Folsomia candida* | 15 °C vs. 20 °C | -0.98 | 0.48 | 0.114 |
|  | 15 °C vs. 25 °C | -2.17 | 0.48 | **<0.001** |
|  | 20 °C vs. 25 °C | -1.19 | 0.48 | **0.043** |
| *Proisotoma minuta* | 15 °C vs. 20 °C | -0.13 | 0.49 | 0.963 |
|  | 15 °C vs. 25 °C | -0.43 | 0.49 | 0.666 |
|  | 20 °C vs. 25 °C | -0.30 | 0.48 | 0.811 |
|  |  |  |  |  |

**Table S9.** Output of the linear model used to assess the effect of the experimental temperature on the mean egg production at first reproductive event of F1 individuals (see Table 1 for the model structure). Estimates, standard errors (SE) and adjusted p-values (*P*) of the contrasts between experimental temperatures for a given species are provided. Significant p*-*values (*P* < 0.05) are highlighted in bold.

**References**

Chahartaghi, M., Scheu, S., Ruess, L., 2006. Sex ratio and mode of reproduction in Collembola of an oak-beech forest. Pedobiologia 50, 331–340. doi:10.1016/j.pedobi.2006.06.001

Fountain, M.T., Hopkin, S.P., 2005. *Folsomia candida* (Collembola): A “standard” soil arthropod. Annual Review of Entomology 50, 201–222. doi:10.1146/annurev.ento.50.071803.130331

Tully, T., 2023. Diversity, plasticity and asynchrony of actuarial and reproductive senescence in the Collembola *Folsomia candida* (Willem, 1902). Frontiers in Ecology and Evolution 11, 1–18. doi:10.3389/fevo.2023.1112045
